# Worldwide Prevalence and Demographic Predictors of Impacted Third Molars—Systematic Review with Meta-Analysis

**DOI:** 10.3390/jcm13247533

**Published:** 2024-12-11

**Authors:** Ana Catarina Pinto, Helena Francisco, Duarte Marques, Jorge N. R. Martins, João Caramês

**Affiliations:** 1 Faculdade de Medicina Dentária, Universidade de Lisboa, 1600-277 Lisboa, Portugal; duarte.marques@campus.ul.pt (D.M.); jnrmartins@edu.ulisboa.pt (J.N.R.M.); carames@campus.ul.pt (J.C.); 2 Implantology Institute, 1070-064 Lisbon, Portugal; 3 LIBPhys-FCT UIDB/04559/2020, Faculdade de Medicina Dentária, Universidade de Lisboa, 1600-277 Lisboa, Portugal; 4 Center for Evidence-Based Dental Medicine, Faculdade de Medicina Dentária, Universidade de Lisboa, 1600-277 Lisboa, Portugal

**Keywords:** demographic predictors, impacted third molars, meta-analysis, prevalence, systematic review

## Abstract

**Background/Objectives:** Impacted teeth are a common phenomenon, in both young and adult populations, as extensively documented in the literature. This study aims to systematically assess the global prevalence of impacted third molars and to analyze their demographic predictors. **Methods:** The present review adheres to PRISMA guidelines and includes studies published until December 2023. Three databases (MEDLINE, LILACS, Cochrane) were searched, and studies reporting prevalence rates and demographic predictors of impacted third molars were selected for analysis. A meta-analysis was conducted using a random-effects model to calculate pooled prevalence and assess demographic variations. **Results:** This review yielded 98 studies involving 183,828 subjects. The pooled prevalence of impacted third molars was 36.9% [95% CI: 33.1–40.7%] per subject and 46.4% [95% CI: 36.7–56.1%] per tooth, with the highest rates in Asia (43.1% [95% CI: 34.6–51.7%]) and the lowest in Europe (24.5% [95% CI: 16.1–33.9%]). An odds ratio of 1.173 [95% CI: 1.021–1.347%] indicated a slightly higher likelihood of third molar impaction among women compared to men, and mandibular third molar impaction was more frequent than maxillary impaction. The meta-regression indicated insufficient consistency for the geographic area to be considered a source of heterogeneity in the prevalence of impacted third molars. **Conclusions:** The present meta-analysis highlights the substantial prevalence of impacted third molars worldwide and underscores the influence of demographic predictors. These findings may inform region-specific clinical guidelines and preventive approaches for managing impacted third molars.

## 1. Introduction

Impacted teeth are a frequent phenomenon in both young and adult populations and are widely reported in the literature [1,2,3,4,5].

According to several studies, mandibular third molars are the most commonly impacted teeth, followed by the maxillary third molars and maxillary canines [1,2,3,6,7].

Despite extensive research on complications, side effects, and surgical best practices, there remains a lack of detailed information on the factors that contribute to the development of M3 impaction. The etiology of third molar impaction remains unclear, with a range of hypothesized causal factors. Among these, the lack of distal physiologic space in the dental arch for third molar eruption (distance from the mandibular ramus to the distal surface of the second molar) has been suggested to be the primary factor contributing to third molar impaction [8,9,10]. Third molar impaction is included in the World Health Organization’s (WHO) International Classification of Diseases (ICD-11) in the group of development diseases [11].

While clinical decision-making is generally straightforward when there are associated pathologies, the prophylactic removal of asymptomatic third molars is still debatable. This ongoing controversy divides the dental community: some advocate for early intervention to mitigate future complications, while others point out the risks associated with extracting asymptomatic teeth [12,13]. This debate has influenced best practice guidelines worldwide, leading to evolving policies over the past decade.

A systematic review conducted in 2016 reported that the global prevalence of impacted third molars in individuals over the age of 17 was 24.4% (95% CI: 18.97% to 30.80%), and the frequency of impaction of one third molar or more ranged between 3.08% and 68.60% across all included studies [14].

There is substantial variability in the reported prevalence rates of impacted third molars, influenced by differences in study design, sampling methods, and population demographics [15,16]. Methodological inconsistencies, such as varying diagnostic criteria and follow-up periods, further complicate cross-study comparisons, contributing to heterogeneity in reported prevalence, and reflect considerable heterogeneity among populations, underscoring the need to better understand the factors driving this variation to assess individual risk more accurately [14,15,17,18].

Understanding these variations is essential for accurately assessing individual risk and refining clinical decision-making. While third molar-related pathologies and management strategies are well documented, the demographic and geographic factors influencing impaction rates remain inadequately explored, with existing research yielding inconsistent results.

Establishing a global pattern of prevalence is an essential step toward uncovering the causes of impaction and understanding its connection to other M3 anomalies, such as agenesis. Furthermore, clarifying how third molar impaction prevalence differs by geographic region, sex, jaw location (mandible or maxilla), and other demographic factors is essential for identifying potential underlying causes. Such insights could enhance best practices in the management of impacted third molars, thereby supporting more informed and effective clinical decision-making.

The present systematic review and meta-analysis aim to assess the global prevalence of impacted third molars through a synthesis of previously published studies and evaluate the influence of demographic factors on these prevalence rates. The null hypothesis to be addressed in the present review was that there are no differences between sexes in the prevalence of impacted third molars. This review was classified as level 4a (systematic review of descriptive studies), providing a comprehensive overview of the epidemiology of impacted third molars across diverse populations.

## 2. Materials and Methods

### 2.1. Protocol and Registration

This systematic review was carried out in compliance with the guidelines outlined in the Preferred Reporting Items for Systematic Reviews and Meta-Analyses (PRISMA) [19,20]. It was registered in the International Prospective Register of Ongoing Systematic Reviews (PROSPERO) in March 2022 under registration number CRD42022311071.

### 2.2. Eligibility Criteria

Inclusion criteria encompassed observational cohort and case–control studies published until 31 December 2023 that evaluated impacted third molars in humans aged 17 years or older. The following exclusion criteria were applied: (1) studies whose corresponding author did not respond to requests for data clarification or manuscript access; (2) studies involving samples that overlapped with other included studies or were based on the same cohort with varying follow-up times; (3) studies that did not report the prevalence of impacted third molars or where prevalence could not be calculated; (4) studies reporting the prevalence of impacted third molars combined with other impacted teeth; (5) studies lacking radiographic evidence to confirm the presence or absence of impacted third molars and their positions; and (6) studies involving patients with orofacial syndromic defects.

### 2.3. Data Source and Search Strategy

A comprehensive literature search was conducted across the following electronic databases: MEDLINE (via PubMed), LILACS, and Cochrane Central Registry of Controlled Trials (CENTRAL). The keywords, MeSH terms, and Boolean connectors used were revised appropriately for each database to account for differences in controlled vocabulary and syntax rules (Table 1). A gray literature search was also conducted in Google Scholar, reviewing the first 300 references, following previously established methods [21]. All identified titles and abstracts were screened for relevance. Additionally, the references of the included articles were manually checked for additional relevant studies. Corresponding authors of potentially relevant studies, or studies requiring data clarification, were contacted via email. Upon successful contact, authors were also queried regarding any additional unpublished or gray literature on the subject. A second search was conducted in August 2024 with a time limit up to 31 December 2023, identifying ten additional articles. The references were managed with a bibliographic software program (Mendeley Reference Manager Version 1.19.8 ©2008–2020).

### 2.4. Study Selection and Data Collection

Study selection was performed in three stages. Firstly, one reviewer (A.C.P.) reviewed all titles and abstracts and selected the relevant ones according to the defined eligibility criteria. Secondly, the full texts of studies deemed relevant were retrieved and assessed against the same eligibility criteria. Thirdly, selected articles underwent critical appraisal to evaluate their scientific rigor and quality.

Two independent reviewers (D.M. and A.C.P.) extracted data from the selected studies. Any disagreements were resolved through discussion or consultation with a third reviewer (H.F.). Data extraction included study characteristics (author, year of publication, country, study design), demographic information (sample size, mean age, sex), and outcome measures (number of patients, number of patients with impacted third molars, total number of evaluated third molars, number of impacted third molars, maxilla/mandible, classification according to Winter’s classification, classification according to Pell and Gregory’s classification, pathology associated with impacted third molar and/or adjacent teeth). Data were extracted from text, tables, or figures, and calculations were performed as necessary. The prevalence of impacted teeth in each study was either directly reported or calculated based on the data provided regarding the relative frequency of impacted teeth and the sample size.

### 2.5. Quality Assessment

The Joanna Briggs Institute Critical Appraisal tools for use in JBI Systematic Reviews—Checklist for Prevalence Studies [22] were used to assess the scientific quality of the included studies. Two reviewers (D.M. and A.C.P.) independently evaluated each study, scoring items as ‘yes’, ‘no’, ‘unclear’, or ‘not applicable’. Discrepancies were resolved by consensus. Cohen’s kappa (k) coefficient ± asymptotic standard error (ASE) was calculated to assess inter-rater agreement for individual questions and overall score [23], as follows: kappa values of <0 indicated less than chance agreement, 0.01–0.20 indicated slight agreement, 0.21–0.40 indicated fair agreement, 0.41–0.60 indicated moderate agreement, 0.61–0.80 indicated substantial agreement, and 0.81–0.99 indicated almost perfect agreement [23]. The overall score for each study, based on the JBI questions, was determined by the percentage of affirmative responses (“yes”). Studies were then classified for risk of bias (RoB) based on their final score as follows: “high” (≤49%), “moderate” (50–69%), or “low” (≥70%) [24].

### 2.6. Statistical Analysis

The study variables were organized and managed in Excel, while statistical analyses were conducted using the OpenMeta [Analyst] v.10.12 software (http://www.cebm.brown.edu/openmeta/ accessed on 24 July 2024). The findings were illustrated through forest plots, displaying untransformed proportions and sex prevalence odds ratios (ORs) along with their respective 95% confidence intervals (CIs) for each study. Additionally, the overall random-effects (Dersimonian–Laird test) pooled estimate and CIs were reported.

The level of statistical heterogeneity among the studies was evaluated using Tau^2^ (τ^2^), which estimates the variance between studies. The Q-Cochran test according to DerSimonian and Laird was applied to detect the presence of heterogeneity, while the I^2^ statistic was used to quantify the extent of heterogeneity for the defined outcomes. The degree of heterogeneity was categorized as low (25%), moderate (50%), or high (75%) [25,26]. Meta-regression was conducted to identify possible sources of between-study heterogeneity in the pooled proportion estimates using geographic regions as explanatory variables. Funnel plot visual analysis was undertaken to assess publication bias using JASP software (JASP 0.19.1, University of Amsterdam, Amsterdam, The Netherlands)) and the regression test for funnel plot asymmetry (Egger’s test) was calculated regarding sex proportions. Statistical significance was set at *p* < 0.05.

## 3. Results

### 3.1. Literature Research and Included Studies

**Figure 1:**
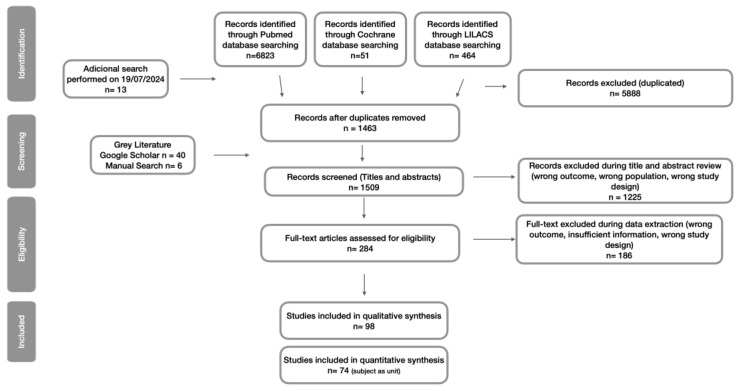
PRISMA flowchart.

The PRISMA flow diagram in Figure 1 outlines the study selection process. The database search yielded a total of 7351 records, with an additional 46 records identified through manual searches and gray literature sources. After duplicate removal, a single reviewer (A.C.P.) screened the title and abstract of the remaining 1463 records. Of these, 284 full-text articles were assessed for eligibility, resulting in 98 studies [2,3,6,7,15,16,17,18,27,28,29,30,31,32,33,34,35,36,37,38,39,40,41,42,43,44,45,46,47,48,49,50,51,52,53,54,55,56,57,58,59,60,61,62,63,64,65,66,67,68,69,70,71,72,73,74,75,76,77,78,79,80,81,82,83,84,85,86,87,88,89,90,91,92,93,94,95,96,97,98,99,100,101,102,103,104,105,106,107,108,109,110,111,112,113,114,115,116,117,118,119] that met the inclusion criteria, encompassing a total of 183,828 subjects (Supplemental Table S1).

### 3.2. Inter-Rater Agreement and Quality Assessment

Two reviewers (D.M. and A.C.P.) independently appraised each eligible study for quality, resolving any discrepancies through discussion until reaching a consensus. JBI items Q5 and Q9 were deemed non-applicable for this analysis. The average Cohen’s kappa coefficient across the 98 studies for inter-rater reliability was 0.83 ± 0.03, indicating an almost perfect agreement. The mean JBI quality score was 63.4% (95% CI: 55.3–71.5%), suggesting a moderate risk of bias (RoB).

### 3.3. Study Characteristics and Synthesis of Results

A summary of the main characteristics of the studies included in the review is provided in Supplemental Table S1. Out of the total 98 studies analyzed, 74 contributed data to the meta-analysis for prevalence per person and 30 studies for prevalence per tooth. The combined prevalence of individuals with at least one impacted third molar was 36.9%, with a 95% confidence interval (CI) of 33.1% to 40.7%. The heterogeneity was notably high (I^2^ = 99.82%, *p* < 0.001), reflecting substantial variability among the studies. This high pooled prevalence and the significant heterogeneity stress the common occurrence of impacted third molars, as well as the diversity in study designs, populations, or definitions across the included studies.

### 3.4. Prevalence of Impacted Third Molars

**Figure 2:**
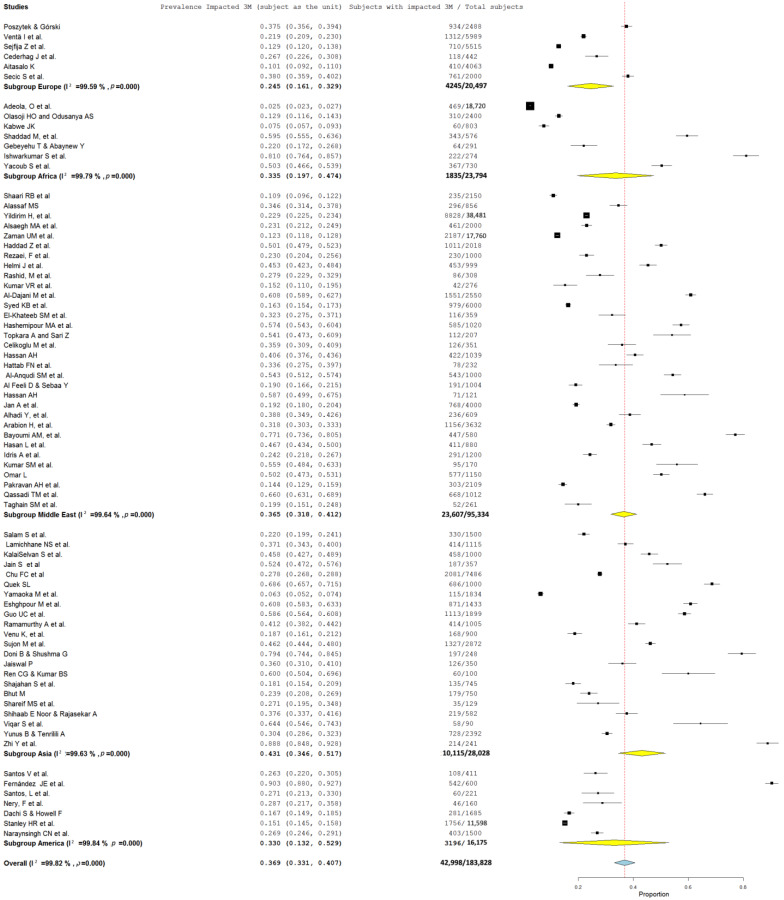
Forest plot of the proportion of subjects with impacted third molars (3Ms) divided by geographic area (subject as the unit).

**Figure 3:**
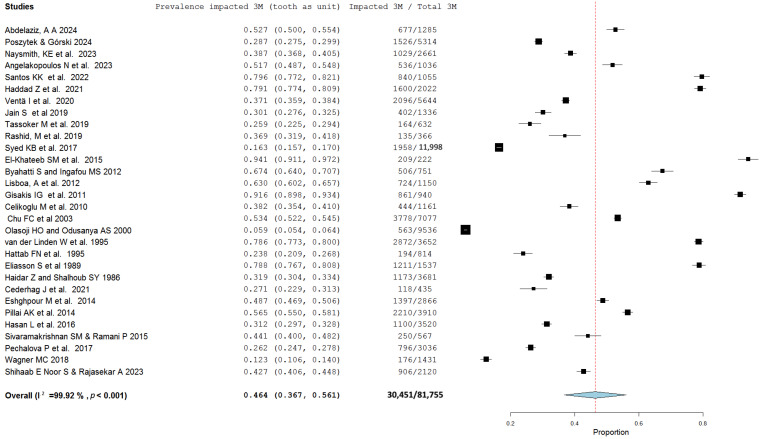
Forest plot of the proportion of impacted third molars (3Ms) (tooth as the unit).

Among the 74 studies included in the meta-analysis to assess for prevalence per person, 36.9% [95% CI: 33.1–40.7%] of subjects had at least one impacted third molar (3M), with significant heterogeneity observed (I^2^ = 99.82%; *p* < 0.001) (Figure 2). When individual third molars (the tooth) were considered as the unit of analysis, 46.4% [95% CI: 36.7–56.1%] were impacted, as reported in 30 studies (I^2^ = 99.92%; *p* < 0.001) (Figure 3).

### 3.5. Geographic Area

The studies provided data from 183,828 patients across various geographic regions. Among these, Asian populations (22 studies; n = 28,028 subjects) exhibited the highest prevalence of impacted third molars at 43.1% [95% CI: 34.6–51.7%], followed by the Middle Eastern populations (32 studies; n = 95,334 subjects) (36.5% [95% CI:31.8–41.2%]). The lowest prevalence was observed in European populations with 24.5% [95% CI: 16.1–32.9%] (6 studies; n = 20,497), while African (7 studies; n = 23,794) and American (7 studies; n = 16,175) populations showed intermediate rates of impaction (33.5% [95% CI:19.7–47.4%] and 33% [95% CI:13.2–52.9%], respectively) (Figure 2).

A forest plot, sub-grouped by geographic region using a random-effects model (Figure 2), revealed substantial heterogeneity (I^2^ = 99.82%) and considerable variability in the frequency of third molar impaction, ranging from 24 to 41%. A meta-regression was conducted to evaluate geographic area as a potential source of heterogeneity in the prevalence of third molar impaction, yielding an omnibus *p*-value of 0.299. This result indicates that the studies included in the meta-analysis do not provide sufficient consistency to attribute geographic area as a definitive source of heterogeneity (*p* > 0.005).

### 3.6. Sex

One study only included male participants [7], while twenty one studies did not report sex distribution for subjects with impacted third molars [6,18,41,51,56,66,67,68,80,81,82,88,101,105,106,109,111,112,113,115,118].

**Figure 4:**
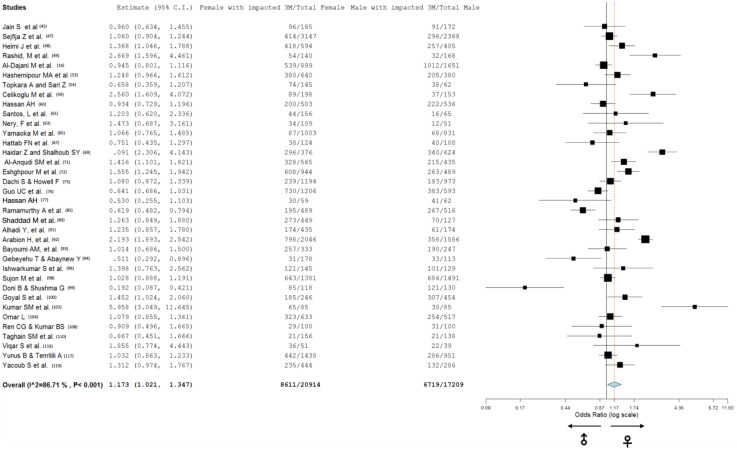
Forest plot of sex distribution in subjects with impacted third molars (3Ms).

**Figure 5:**
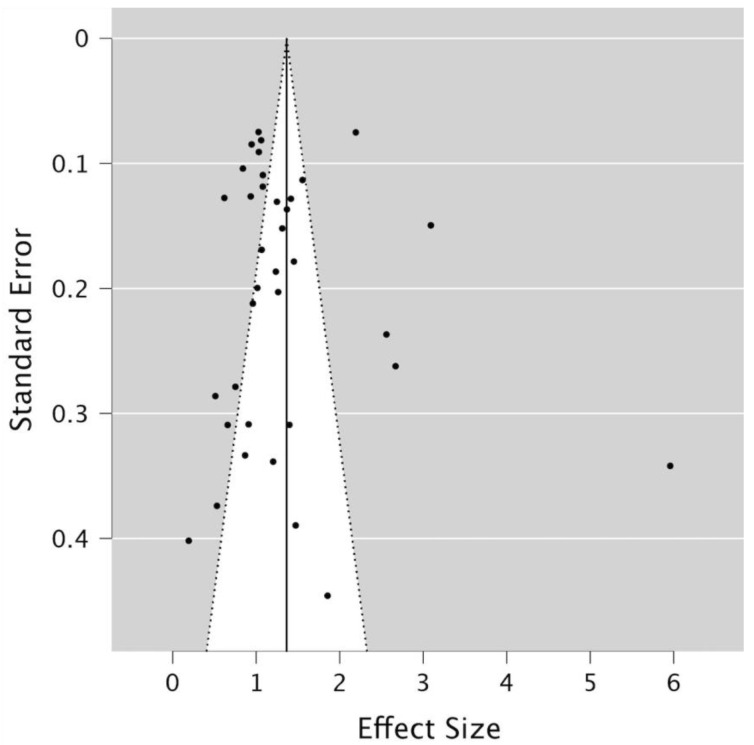
Funnel plot visual analysis and regression test for funnel plot asymmetry (Egger’s test). Each dot correspond to a study included in this systematic review.

Among the 35 studies that provided sex-specific data, a total of 20,914 women were included, of whom 8611 had at least one impacted third molar. In contrast, 17,209 males were included, with 6719 exhibiting at least one impacted third molar. An odds ratio of 1.173 indicated a slightly higher likelihood of third molar impaction among women compared to men (Figure 4). Furthermore, Egger’s test was applied to assess sex proportion as a potential source of publication bias (z = 0.56; *p* = 0.58), suggesting no strong evidence of bias (Figure 5).

### 3.7. Dental Arch

Eighteen studies evaluated impacted third molars in the mandibular arch exclusively, while twenty-seven studies did not specify the distribution of impacted third molars by dental arch. Of the 53 studies that reported impacted third molar distribution according dental arch, most identified a higher prevalence in the mandible, and only 4 indicated a slight predominance in the maxilla [27,34,65,110].

## 4. Discussion

### 4.1. Key Findings and Clinical Implications

This systematic review and meta-analysis comprehensively assess the global prevalence of impacted third molars, revealing a pooled prevalence of 36.9% among subjects and 46.4% at the tooth level. These findings highlight a significant burden across diverse populations and indicate impacted third molars as a persistent dental issue. Notably, regional variation in prevalence was substantial, with the highest rates observed in Asia (43.1%) and the Middle East (36.5%), and the lowest in Europe (24.5%). This variation indicates that geographic and possibly environmental or genetic factors may influence the likelihood of impaction. Given the clinical complexities associated with impacted third molars (e.g., infection, pain, and potential damage to adjacent structures), these prevalence rates emphasize the need for region-specific management strategies and preventative approaches, especially in high-prevalence regions.

Previous studies have suggested that third molar eruption is generally completed between 17 and 20 years of age [6,76]. Therefore, 17 years was selected as the minimum age in the inclusion criteria.

With regard to sex distribution, our results suggest a slightly higher prevalence of impacted third molars in females than in males, with an odds ratio of 1.173. This finding aligns with the previous literature suggesting that anatomical differences, such as smaller jaw dimensions in females, may influence impaction rates [54,64,76,77,85,94,102,110]. Additionally, the delayed or limited mandibular growth in females, particularly around the onset of menarche, may exacerbate impaction risk, whereas continued mandibular growth in males until eruption age potentially mitigates impaction [48,50,64,77]. Environmental influences, including health status, socioeconomic factors, and energy balance associated with physical activity, could also contribute to this sex disparity. The variability in menarche timing across populations might further explain the observed sex differences in the prevalence of third molar impaction [77,94].

Additionally, the higher prevalence of mandibular impactions found in this review aligns with earlier studies and supports clinical observations that mandibular third molars encounter more spatial limitations, increasing the likelihood of impaction [53,84,94,98,119].

### 4.2. Strengths and Limitations of the Evidence

This review presents several strengths, including a thorough search conducted across multiple databases and gray literature sources, which helped to reduce the likelihood of publication selection bias. Furthermore, the systematic approach to data extraction and quality assessment ensured that only studies meeting the established criteria for rigor were included, enhancing the robustness of the findings.

At the review level, certain limitations should be acknowledged when interpreting the results of this meta-analysis. First, the heterogeneity across studies reduces the reliability of the pooled prevalence estimates. Additionally, many studies did not control for confounding factors, such as socioeconomic status and dietary influences, which could affect third molar impaction rates. The reliance on observational data further introduces potential biases, including selection and reporting biases, which may skew prevalence estimates. Furthermore, the exclusion of studies lacking radiographic evidence and the reliance on cross-sectional designs limit insights into the natural progression of impactions and potential etiological factors.

At the level of outcomes, it is important to account for certain limitations. The demographic focus of this review, which included studies primarily from Asia, the Middle East, and Europe, limits the generalizability of these findings to other regions.

### 4.3. Comparison with Previous Literature

This review expands significantly on the findings of the previous systematic review on this topic, incorporating nearly three times as many subjects (n = 183,828) [14]. The findings of this review are consistent with previous studies reporting a global high prevalence of impacted third molars. However, this review expands on prior research by comprehensively analyzing demographic predictors and regional differences. The higher prevalence in Asian populations aligns with previous findings, which have suggested that dietary and genetic factors might influence mandibular development and increase impaction rates [39,64,89]. The sex differences observed are consistent with reports suggesting a higher prevalence of third molar impaction in females due to the different developmental growth profile patterns between males and females but also the fact that jaw growth in females typically stops at the time when third molars start to erupt, whereas in males the growth continues during the period of eruption, providing adequate space for third molar eruption [48,50]. This review also reinforces the predominance of mandibular impactions, likely due to anatomical constraints in the mandibular arch.

### 4.4. Clinical Impact and Recommendations

The findings of this review have significant clinical implications. The high prevalence of impacted third molars, particularly in Asia and the Middle East, indicates a need for region-specific guidelines considering the unique demographic and anatomical factors contributing to impaction. Additionally, given the slightly higher prevalence in females, targeted screening practices could benefit this group, particularly during the late teenage years when impactions are often first detectable. The proactive identification of patients at higher risk may aid in early intervention and reduce the potential for complications, such as pericoronitis, cyst formation, or damage to adjacent teeth.

The predominance of mandibular impactions further suggests that clinicians should pay particular attention to the mandibular region when assessing the third molar eruption potential. The lack of space in the retromolar region, which may result from insufficient mandibular growth or a discrepancy between tooth and jaw sizes, seems to be the primary factor contributing to third molar impaction. Additionally, a narrow alveolar arch and delayed third molar maturation combined with early physical maturation may also contribute to impaction [77].

The management of impacted third molars is a constant decision present in the daily clinical practice of an oral surgeon. It is estimated that more than 50% of the population experience problems with their third molars, including problems with the eruption process or other pathologies [120]. While the decision-making is generally straightforward when there are associated pathologies, controversy persists with respect to removing asymptomatic third molars instead of regular long-term follow-ups. First, it is important to state that being asymptomatic is not synonymous of being disease-free but solely the absence of symptoms. Occasionally, the disease can progress without symptoms, so this should also be taken into account in the management of third molars [10,121]. Considering the complications that may occur in the presence of impacted third molars, a careful assessment of the tooth position and its eruption status is mandatory to optimize treatment.

While impacted third molars are more frequently removed in younger patients due to pericoronitis, caries are a more common reason for extraction in older patients [122]. Many unerupted teeth are removed in younger people, though they are not yet determined to be impacted, but their removal may be justifiable for orthodontic purposes, whether contributing to maintain the results of the orthodontic treatment or when it is necessary to move distally the posterior sector of the dental arch or when preventing the eruption of the second molar [10,123,124]. Considering the surgical complexities and associated risks of removing mandibular impacted third molars, preventive or conservative approaches may be warranted, especially for asymptomatic cases that may not require routine extraction, mainly in older patients.

Future regional guidelines should reflect these considerations, aiming to balance the risks of early intervention with the potential for disease progression in untreated impacted third molars.

## 5. Conclusions and Future Research Directions

The present review provides a comprehensive overview of impacted third molars’ prevalence, highlighting substantial geographic and demographic variations. The findings suggest that impacted third molars are a prevalent condition worldwide, with implications for public health and clinical practice. Future studies should address the limitations identified in this review, particularly through longitudinal studies that can better elucidate the natural progression of impacted third molars. Additionally, studies employing standardized diagnostic criteria and including a broader geographic range will enhance the generalizability and reliability of the findings. More balanced geographic representation, as well as studies with standardized protocols, would improve the accuracy and applicability of future research in this area. Ultimately, these efforts may inform more effective and tailored management strategies for impacted third molars.

## Figures and Tables

**Table 1:** Terms used in each electronic database.

Database	Terms Used	Filters
MEDLINE (via PubMed)	(“tooth, impacted” [MeSH] OR “impacted tooth” OR “teeth, impacted” OR “impacted teeth” OR “cuspid” [MeSH] OR “canine” OR “bicuspid” [MeSH] OR “premolar” OR “premolars” OR “molar, third” [MeSH] OR “third molars” OR “wisdom teeth” OR “wisdom tooth”) AND (“impaction” OR “impacted” OR “retained”) AND (“prevalence” [MeSH] OR “frequency”)	Publication date: 1980–2023
Lilacs	((impacted tooth) OR (impacted teeth)) AND ((impacted) OR (retained)) AND ((prevalence) OR (frequency))	Publication date: 1980–2023
Cochrane Collaboration	“impacted teeth”	Publication date: 1980–2023

## Data Availability

The original contributions presented in the study are included in the article, further inquiries can be directed to the corresponding authors.

## Supplementary Materials

The following supporting information can be downloaded at: https://www.mdpi.com/article/10.3390/jcm13247533/s1, Supplemental Table S1: Included articles.
