# Supplementary material for: Worldwide Prevalence and Demographic Predictors of Impacted Third Molars—Systematic Review with Meta-Analysis
Source: J Clin Med. 2024 Dec 11;13(24):7533. doi: 10.3390/jcm13247533 (PMC11728092; doi:10.3390/jcm13247533)
Supplement: Supplementary file 1 [file jcm-13-07533-s001.zip › jcm-3349966-supplementary.pdf]

						Subjects as unit		
**Author**	**Title**	**Year**	**Country**	**Journal**	**Study design**	**N Total patients**	**N patients with impacted 3M**	**Prevalence patients with impacted 3M**
Abdelaziz, A A	Prevalence of impacted third molars among different sagittal skeletal relationships in Upper Egyptian patients	2024	Egypt	Egyptian Dental Journal	Retrospective	352	352	-
Poszytek & Górski	Relationship between the Status of Third Molars and the Occurrence of Dental and Periodontal Lesions in Adjacent Second Molars in the Polish Population: A Radiological Retrospective Observational Study	2024	Poland	J. Clin. Med	Retrospective	2488	934	37.5% (631 (25.23%) had at least one partially impacted 3M, and 303 participants (12.18%) had at least one completely impacted 3M)
Adeola, O et al.	Impacted Mandibular Third Molar Prevalence and Patterns in a Nigerian Teaching Hospital: A 5-Year Retrospective Study	2023	Nigeria	Biomed	Retrospective	18720	469	2.5%
Naysmith, KE et al.	Oral Health Status of New Zealand Defence Force Recruits.	2023	New Zealand	Military medicine	Cross-sectional	874	-	-
Shaari RB et al.	Prevalence and pattern of third molars impaction: A retrospective radiographic study.	2023	Iraq	Journal of advanced pharmaceutical technology & research	Retrospective	2150	235	10.9%
Salam S et al.	Prevalence of Impacted Teeth and Pattern of Third Molar Impaction among Kerala Population a Cross Sectional Study.	2023	India	Journal of pharmacy & bioallied sciences	Cross-sectional	1500	330	22%

Angelakopoulos N et al.	Effect of impaction on third molar development and age estimation-A study in a Lebanese population.	2023	Lebano	Morphologie : bulletin de l'Association des anatomistes	Retrospective	518			
Alassaf MS	Assessment of the Relation Between Patterns of Third-Molar Impaction and Blood Group: A Retrospective Study.	2023	Arabia Saudita	Cureus	Cross-sectional	856	296	34.6%	
Lamichhane NS et al.	Mandibular Second Molars and Their Pathologies Related to the Position of the Mandibular Third Molar: A Radiographic Study.	2023	Nepal	Clinical, cosmetic and investigational dentistry	Cross-sectional	1115	414	37.1%	
Yildirim H, et al.	Investigation of the prevalence of impacted third molars and the effects of eruption level and angulation on caries development by panoramic radiographs.	2022	Turkey	Med Oral Patol Oral Cir Bucal.	Cross-sectional	38481	8828 (had at least 1 impacted 3M)	22.9%	
Alsaegh MA et al.	The pattern of mandibular third molar impaction and its relationship with the development of distal caries in adjacent second molars among Emiratis: a retrospective study.	2022	UAE	BMC oral health	Retrospective	2000	461	23.05%	

Santos KK et al.	Prevalence of Mandibular Third Molars According to the Pell & Gregory and Winter Classifications.	2022	Brazil	Journal of maxillofacial and oral surgery	Retrospective			
Ahmad P et al.	Pattern of third molar impactions in north-eastern peninsular Malaysia: A 10-year retrospective study.	2021	Malaysia	Nigerian journal of clinical practice	Retrospective	490		
Zaman UM et al.	Pattern of Mandibular Third Molar Impaction in Nonsyndromic 17760 Patients: A Retrospective Study among Saudi Population in Central Region, Saudi Arabia.	2021	Arabia Saudita	BioMed research international	Retrospective	17760	2187 (1337 bilateral; 850 unilateral)	12.31%
Haddad Z et al.	Radiographic Position of Impacted Mandibular Third Molars and Their Association with Pathological Conditions.	2021	Iran	International journal of dentistry	Retrospective	2018	1011 (589 bilateral)	50.10%
Ventä I et al.	Prevalence of third molars determined by panoramic radiographs in a population-based survey of adult Finns.	2020	Finland	Community dentistry and oral epidemiology	Cross-sectional	5989 (5644 3M)		At least one impacted third molar was found in 21.9% of the population
KalaiSelvan S et al.	Prevalence and Pattern of Impacted Mandibular Third Molar: An Institution-based Retrospective Study.	2020	India	Journal of pharmacy & bioallied sciences	Retrospective	1000	458	45.8%
Rezaei, F et al.	Patterns of Mandibular Third Molar Impaction in an Iranian Subpopulation	2020	Iran	Pesqui. bras. odontopediatria clín. Integr	Retrospective	1000	230	23%

Jain S et al.	Prevalence of impacted third molars among orthodontic patients in different malocclusions.	2019	India	Indian journal of dental research	Retrospective	357	187	52.38%
Tassoker M et al.	Is There a Possible Association between Skeletal Face Types and Third Molar Impaction? A Retrospective Radiographic Study.	2019	Turkey	Medical principles and practice : international journal of the Kuwait University	Retrospective	158		
Sejfija Z et al.	Prevalence of Pathologies Associated with Impacted Third Molars in Kosovar Population: an Orthopanthomography Study.	2019	Croatia	Acta stomatologica Croatica	Retrospective	5515	710 (≥ 1 3M impactado)	12.87%
Helmi J et al.	Prevalence of impacted teeth among a sample of yemeni population and their association with sex and age	2019	Yemen	J. oral res. (Impresa)	Cross-sectional	999	453	45.35%
Rashid, M et al.	Prevalence of impacted mandibular third molars and its association with distal caries in mandibular second molars using cone beam computed tomography	2019	Iraq	J. oral res. (Impresa)	Retrospective	308	86	27.92%
Kumar VR et al.	Prevalence and Pattern of Mandibular Third Molar Impaction in Eritrean Population: A Retrospective Study.	2017	Saudi Arabia	The journal of contemporary dental practice	Retrospective	276	42	15.2%
Al-Dajani M et al.	A Cohort Study of the Patterns of Third Molar Impaction in Panoramic Radiographs in Saudi Population.	2017	Saudi Arabia	The open dentistry journal	Retrospective	2550	1551	60.82%
Syed KB et al.	Prevalence of Distal Caries in Mandibular Second Molar Due to Impacted Third Molar.	2017	**Saudi Arabia**	Journal of clinical and diagnostic research : JCDR	Retrospective	6000	979	16.27%

El-Khateeb SM et al.	Radiographic assessment of impacted teeth and associated pathosis prevalence. Pattern of occurrence at different ages in Saudi male in Western Saudi Arabia.	2015	Saudi Arabia	Saudi medical journal	Retrospective	359	116	40.67%
Chicarelli da Silva, M et al.	Estudios radiográfico de la prevalencia de impactaciones dentarias de terceros molares y sus respectivas posiciones	2014	Brazil	Acta odontol. Venez	Retrospective	1004	279	
Hashemipour MA et al.	Incidence of impacted mandibular and maxillary third molars: a radiographic study in a Southeast Iran population.	2013	Iran	Medicina oral, patologia oral y cirugia bucal	Retrospective	1020	585 (pelo menos 1 3M impactado)	57.35%
Topkara A and Sari Z	Investigation of third molar impaction in Turkish orthodontic patients: Prevalence, depth and angular positions.	2013	Turkey	European journal of dentistry	Retrospective	207	112	54.11%
Santos V et al.	Prevalência radiográfica de dentes não irrompidos e supranumerários	2013	Brazil	RGO (Porto Alegre)	Retrospective	411	108	26.28%
Byahatti S and Ingafou MS	Prevalence of eruption status of third molars in Libyan students.	2012	Libya	Dental research journal	Retrospective	200		
Lisboa, A et al.	Prevalência de inclinações e profundidade de terceiros molares inferiores, segundo as classificações de winter e de pell & gregory	2012	Brazil	Pesqui. bras. odontopediatria clín. Integr	Retrospective	575		

Gisakis IG et al.	Prevalence of impacted teeth in a Greek population.	2011	Greece	Journal of investigative and clinical dentistry	Retrospective	425		
Fernández JE et al.	Estudio estadístico de la prevalencia de 3° molares inferiores retenidos en radiografías panorámicas	2011	Argentina	Rev. arg. Morfol	Retrospective	600	542	90,33%
Celikoglu M et al.	Frequency of agenesis, impaction, angulation, and related pathologic changes of third molar teeth in orthodontic patients.	2010	Turkey	Journal of oral and maxillofacial surgery	Retrospective	351	126	35.9%
Hassan AH	Pattern of third molar impaction in a Saudi population.	2010	Saudi Arabia	Clinical, cosmetic and investigational dentistry	Retrospective	1039	422	40.5%
Santos, L et al.	Análise radiográfica da prevalência de terceiros molares retidos efetuada na clínica de odontologia do Centro Universitário Positivo	2006	Brazil	RSBO (Impr.)	Retrospective	221	60	27.15%
Nery, F et al.	Avaliação da prevalência de terceiros molares inferiores inclusos e da posição e inclinação do seu longo eixo em radiografias panorâmicas	2006	Brazil	Rev. Ciênc. Méd. Biol. (Impr.)	Retrospective	160	46	28.75%
Chu FC et al.	Prevalence of impacted teeth and associated pathologies--a radiographic study of the Hong Kong Chinese population.	2003	Hong Kong	Hong Kong medical journal	Retrospective	7486	2081 (pelo menos 1)	27.8%
Quek SL	Pattern of third molar impaction in a Singapore Chinese population: a retrospective radiographic survey.	2003	singapore	International journal of oral and maxillofacial surgery	Retrospective	1000	686	6.86%

Olasoji HO and Odusanya AS	Comparative study of third molar impaction in rural and urban areas of South-Western Nigeria.	2000	Nigeria	Odonto-stomatologie tropicale	Retrospective	2400	310	12.92%
Yamaoka M et al.	Influence of adjacent teeth on impacted third molars in the upper and lower jaws.	1995	Japan	Australian dental journal	Retrospective	1834	115	6.63%
van der Linden W et al.	Diseases and lesions associated with third molars. Review of 1001 cases.	1995	South Africa	Oral surgery, oral medicine, oral pathology, oral radiology, and endodontics	Retrospective	1001		
Hattab FN et al.	Impaction status of third molars in Jordanian students.	1995	Jordania	Oral surgery, oral medicine, oral pathology, oral radiology, and endodontics	Retrospective	232	78 (pelo menos um)	33.62%
Eliasson S et al.	Pathological changes related to long-term impaction of third molars. A radiographic study.	1989	Sweden	International journal of oral and maxillofacial surgery	Retrospective	644		
Haidar Z and Shalhoub SY	The incidence of impacted wisdom teeth in a Saudi community.	1986	Saudi Arabia	International journal of oral and maxillofacial surgery	Retrospective	1000		

Cederhag J et al.	Evaluation of Panoramic Radiographs in Relation to the Mandibular Third Molar and to Incidental Findings in an Adult Population.	2021	Sweden	European journal of dentistry	Retrospective	442	118	26.70%
Al-Anqudi SM et al.	Prevalence and Pattern of Third Molar Impaction: A retrospective study of radiographs in Oman.	2014	Oman	Sultan Qaboos University medical journal	Retrospective	1000	543	
Eshghpour M et al.	Pattern of mandibular third molar impaction: A cross-sectional study in northeast of Iran.	2014	India	Nigerian journal of clinical practice	Retrospective	1433	871	
Aitasalo K	An orthopantomographic study of prevalence of impacted teeth	1972	Finland	Int. J. oral Surg.	Retrospective	4063	410	70.2%
Al Feeli D & Sebaa Y	Prevalence of Impacted Teeth in Adult Patients: A Radiographic Study of Kuwaiti Population	2013	Kuwaiti	Thesis	Retrospective	1004	191	
Dachi S & Howell F	survey of 3,874 routine full-mouth radiographs: II. A study of impacted teeth.	1961	USA	Oral Surg Oral Med Oral Pathol.	Retrospective	1685	281	
Guo UC et al.	The influence of impaction to the third molar mineralization in northwestern Chinese population.	2014	China	Int J Legal Med	Retrospective	1899	1113	
Hassan AH	Mandibular cephalometric characteristics of a Saudi sample of patients having impacted third molars.	2011	Saudi Arabia	Saudi Dent J.	Retrospective	121	71	

Hatem M et al.	Pattern of third molar impaction in Libyan population: a retrospective radiographic study	2016	Libya	Saudi J Dent Res.	Retrospective	300		
Jan A et al.	Prevalence of impacted third molars in Jeddah, Saudi Arabia: a retrospective study	2014	Saudi Arabia	Journal of American Science	Retrospective	4000	768	
Jung YH & Cho BH	Prevalence of missing and impacted third molars in adults aged 25 years and above	2013	Korea	Imaging Science in Dentistry	Retrospective	3799		
Kabwe JK	THE DISTRIBUTION OF DENTAL DISEASES AND DISORDERS OBSERVED IN NA URBAN DENTAL CLINIC IN ZAMBIA	1996	Zambia	Trop Dent J.	Retrospective	803	60	
Kruger E et al.	Third molar outcomes from age 18 to 26: Findings from a population-based New Zealand longitudinal study	2001	New Zealand	Oral Surg Oral Med Oral Pathol Oral Radiol Endod	Retrospective	842		
Perumal CJ	A Comparative Pan-Oral Radiographic Survey of Third Molars in Black and In Indian South Africans	2013	South Africa	Perumal CJ. Dentistry Adv Res	Retrospective	1128 (564 black; 564 indian)		
Pillai AK et al.	Incidence of impacted third molars: a radiographic study in People’s Hospital Bhopal India	2014	India	J Oral Biol Craniofac Res.	Retrospective	1100		
Ramamurthy A et al.	Prevalence of mandibular third molar impaction and agenesis: a radiographic south Indian study	2012	India	J Ind Aca Oral Med Radiol.	Retrospective	1005	414	

Venu K, et al.	Distribution of Third Molar Impactions Among Rural and Urban Dwellers in the Age Group of 22–30 years in South India: A Comparative Study	2012	India	J. Maxillofac. Oral Surg.	Observational	900	168	
Secic S et al.	Incidence of impacted mandibular third molars in population of Bosnia and Herzegovina: a retrospective radiographic study.	2013	Bosnia and Herzegovin a	J Health Sci.	Retrospective	2000	761	
Stanley HR et al.	Pathological sequelae of “neglected” impacted third molars.	1988	USA	J Oral Pathol Med.	Retrospective	11598	1756	
Shaddad, M et al.	Prevalence and Pattern of Impacted Third Molar in a Sample of Sudanese Population	2018	Sudan	Indian Journal of Dental Education	Retrospective	576	343	
Alfadil L & Almajed E	Prevalence of impacted third molars and the reason for extraction in Saudi Arabia	2020	Saudi Arabia	Saudi Dent J.	Retrospective	1014		
Alhadi Y, et al.	Prevalence and Pattern of Third Molar Impaction in Sample of Yemeni Adults	2019	Yemen	Online Journal of Dentistry & Oral Health	Retrospective	609	236	
Arabion H, et al.	Prevalence of Impacted Teeth among Young Adults: A Retrospective Radiographic Study	2017	Iran	Dent Mater Tech	Retrospective	3632	1156	
Bayoumi AM, et al.	The Prevalence Rate of Third Molar Impaction among Jeddah Population	2016	Saudi Arabia	International Journal of Dentistry and Oral Health	Retrospective	580	447	

Gebeyehu T & Abaynew Y	Prevalence and patterns of third molar impaction among Ethiopians in Addis Ababa: a retrospective pilot study	2024	Ethiopia	Nature portfolio	Retrospective	291	64	
Hasan L et al.	Impacted wisdom teeth, prevalence, pattern of impaction, complications and indication for extraction: A pilot clinic study in Iraqi population	2016	Iraq	Tikrit Journal for Dental Sciences	Retrospective	880	411	
Idris A et al.	Third molar impaction in the Jazan Region: Evaluation of the prevalence and clinical presentation	2021	Saudi Arabia	Saudi Dental Journal	Retrospective	1200	291	
Ishwarkumar S et al.	Prevalence of impacted third molars in the South African Indian population of the eThekwini Metropolitan Region	2019	South Africa	SADJ	Retrospective	274	222	
Sivaramakrishnan SM & Ramani P	Study on the Prevalence of Eruption Status of Third Molars in South Indian Population	2015	India	Biol Med (Aligarh)	Retrospective	150		
Sujon M et al.	Third Molar Impactions Prevalence and Pattern Among Adults Using 5923 Digital Orthopantomogram	2022	Bangladesh	Bangladesh Journal of Medical Science	Retrospective	2872	1327	
Doni B & Shushma G	Assessment of prevalence and pattern of impacted third molar among known population: A retrospective analysis	2019	India	International Journal of Applied Dental Sciences	Retrospective	248	197	
Goyal S et al.	Radiographic Evaluation of the Status of Third Molars in Sriganganagar Population – A Digital Panoramic Study	2016	India	Malays J Med Sci.	Retrospective	700		

Jaiswal P	Prevalence of eruption status of wisdom teeth (Third molar) in Rajnandgaon (Chhattisgarh) population: A digital panoramic study	2018	India	International Journal of Applied Dental Sciences	Retrospective	350	126	
Kumar SM et al.	Distribution of Impacted Third Molars based on Gender and Patterns of Angulation in Dental Students of the Hai'l Region, Saudi Arabia: A Panoramic Radiographic (OPG) Study	2017	Saudi Arabia	International Journal of Contemporary Medical Research	Retrospective	170	95	
Naraynsingh CN et al.	Prevalence and Pattern of Tooth Impaction: A Radiographic Study in a Trinidadian Population	2024	Caraíbas	Nigerian Journal of Clinical Practice	Retrospective	1500	403	
Omar L	Prevalence of Impacted Wisdom Teeth among Hawler Young People	2008	Iraq	MDJ	Retrospective	1150	577	
Pakravan AH et al.	Evaluation of impact teeth prevalence and related pathologic lesions in patients in Northern part of Iran (2014-2016)	2018	Iran	J Contemp Med Sci	Retrospective	2109	303	
Pechalova P et al.	Mandibular Third Molars in Southern Bulgaria – A Clinical and Radiological Study of 1518 Patients	2017	Bulgaria	Journal of Dental Problems and Solutions	Observational	1518		
Qassadi TM et al.	Prevalence and Pattern of Third Molar Impaction among the Saudi Population in Jazan Region, Saudi Arabia	2020	Saudi Arabia	Saudi Journal of Oral and Dental Research	Retrospective	1012	668	
Ren CG & Kumar BS	Prevalence of Eruption of Third Molar Tooth among South Indians and Malaysians	2014	India	Journal of Academy of Dental Education,	Retrospective	100	60	

Shajahan S et al.	Prevalence and Pattern of Distribution of Impacted Mandibular Third Molars on Digital Panoramic Radiograph among Central Kerala Population: A Cross Sectional Study	2019	India	Journal of medical science and clinical research	Retrospective	745	135	
Taghain SM et al.	The Prevalence of Impacted Third Molar, Impaction Angulation, and Impaction Depth in Patients Visiting Dental Clinics and Private Offices in Ghaemshahr, Iran, in 2016	2022	Iran	Journal of Research in Applied and Basic Medical Sciences	Retrospective	261	52	
Wagner MC	Epidemiological Survey of third molars in individuals from the city of Bauru	2018	Brazil	Thesis	Retrospective	500		
Bhut M	Prevalence of impacted tooth: A radiographic study of 750 cases	2022		European Journal of Molecular & Clinical Medicine		750	179	
Genc B et al.	Maxillary and Mandibular Third Molars Impaction with Associated Pathologies in a North Cyprus Population: A Retrospective Study	2022	Cyprus	Applied Sciences	Retrospective	550		
Shareif MS et al.	Pattern of Mandibular Third Molar Impaction in Malaysia Population and Their Association with Gender, Age and Race	2020	Malaysia	Journal of International Dental and Medical Research	Retrospective	129	35	
SHIHAAB E NOOR S & RAJASEKAR A	PREVALENCE OF THIRD MOLAR TEETH IMPACTION IN SOUTH INDIAN POPULATION. A RADIOGRAPHIC ASSESSMENT	2023	India	Oral Radiology	Retrospective	582	219	
Viqar S et al.	The Frequency of Mandibular Third Molar Impaction in Different Types of Vertical Skeletal Faces	2021	Pakistan	J Pak Dent Assoc	Retrospective	90	58	

Yunus B & Tenrilili A	Prevalence of impacted third molar during covid-19 pandemic at Hasanuddin University Dental Hospital	2023	Indonesia	Makassar Dental Journal	Retrospective	2392	728	
Zhi Y et al.	Prevalence of Impacted Third Molars by Winter's Classification and Pell & Gregory's Classification on Radiographic Assessment in Relation to ABO Blood Group in Orthodontic Patients in Hospital Universiti Sains Malaysia (HUSM)	2019	Malaysia	International Medical Journal	Retrospective	241	214	
Yacoub S et al.	Prevalence of impacted third molars: A radiographic study among a North African population	2024	Tunisia	La Tunisie Medicale	Retrospective	730	367	

	Teeth as unit							
Author	N 3M	N missing 3M	Total impacted 3M	Prevalence impacted 3M (teeth)	N MAXILLA impacted 3M	N MANDIBLE impacted 3M	Gender	Age
Abdelaziz, A A	1285	123	677	52,68%	340	337	MaleImpacted:163 FemaleImpacted: 189	18-40y
Poszytek & Górski	5314		1526	28.7%			MaleTotal:1055 FemaleTotal: 1433	42.2y
Adeola, O et al.	-	-	-	-			MaleImpacted:180 FemaleImpacted: 289	20–29: 343 30-39: 101 40-49: 16 50-73: 9
Naysmith, KE et al.	2661	684	1029	38,67%	227	802	MaleTotal:701 FemaleTotal: 173	17-18: 310 19-20: 237 21-24: 225 ≥25: 102
Shaari RB et al.			473	34.71% (51.49% 1 impacted 3M; 16.6% 2 impacted 3M; 11.06% 3 impacted 3M; 20.85% 4 impacted 3M)	188	285	MaleTotal:359 FemaleTotal: 318 Male Impacted: 112 Female Impacted: 123	21-30: 422 31-40: 255
Salam S et al.			593	-	105	488	MaleTotal:816 FemaleTotal: 684 Mela Impacted: 176 Female Impacted: 154	30.15 ± 5.75y

Angelakopoulos N et al.	1036		536	51.7%	only mandible	536	MaleTotal:289<br>FemaleTotal: 229	Median 18y
Alassaf MS			672		387	285	MaleTotal:739<br>FemaleTotal: 117	18-85y (34.78 (±13.68))
Lamichhane NS et al.			758		only mandible	414	MaleImpacted:247<br>FemaleImpacted: 167	20-73y (37.90±13.85)
Yildirim H, et al.			18331	-	7648	10683	impacted 3M: 55.3% females; 44.7% males	> 25y
Alsaegh MA et al.			686	-	only mandible	686	Male Impacted: 253<br>Female Impacted: 433	18-51y (26.24)

Santos KK et al.	1055		840	79.6%	only mandible	840	MaleTotalteeth: 459<br>FemaleTotalteeth: 596	26.77y
Ahmad P et al.	1960		1957		935	1022	MaleTotal: 153<br>FemaleTotal: 337<br>MaleImpacted:678<br>FemaleImpacted: 1279	20-64y (28.87 ± 6.23 years)
Zaman UM et al.					only mandible	2187	MaleImpacted:1065<br>FemaleImpacted: 1788	
Haddad Z et al.	2022		1600	79.13%	only mandible	1600		20-55y (25.73 ± 5.26)
Ventä I et al.	5644		2096 (886 soft tissue; 1210 bone)	37.14%	606	1490	MaleTotal: 2749<br>FemaleTotal: 3240	52.5y
KalaiSelvan S et al.					only mandible		MaleImpacted: 260<br>FemaleImpacted: 198	20-40y (30.5y (with I3M)
Rezaei, F et al.			350		only mandible	350	MaleTotal: 92<br>FemaleTotal: 138	18-58y (32.8 ± 8.3 years)

Jain S et al.	1336	92	402	30.1%	169	233	MaleTotal: 172<br>FemaleTotal: 185<br>MaleImpacted:91<br>FemaleImpacted: 96	
Tassoker M et al.	632	0	164	25.95%	122	206	MaleTotal: 61<br>FemaleTotal: 97	19-25y (20.19 ± 1.62)
Sejfija Z et al.			1297				MaleTotal: 2368<br>FemaleTotal:3147<br>MaleImpacted: 296<br>FemaleImpacted: 414	18-77y (29.3 ± 12.8).)
Helmi J et al.			675		303	372	MaleTotal: 405<br>FemaleTotal: 594	17-54y (26.6y)
Rashid, M et al.	366		135	36.88%	only mandible	135	MaleTotal: 168<br>FemaleTotal: 140	30.28±10y
Kumar VR et al.					only mandible		MaleTotal: 129<br>FemaleTotal: 147	30±6.839y
Al-Dajani M et al.			2650		726	825	MaleTotal: 1651<br>FemaleTotal: 899<br>MaleImpacted: 1012<br>FemaleImpacted: 539	35.8y
Syed KB et al.	11998		1958	16.32%	only mandible			

El-Khateeb SM et al.			209		66	143	All male	
Chicarelli da Silva, M et al.			664		245	419	MaleTotal: 110<br>FemaleTotal: 169	23.29y
Hashemipour MA et al.			1165		415	750	MaleTotal: 380<br>FemaleTotal: 640	26.2±5.8y
Topkara A and Sari Z			300		148	152	MaleTotal: 62<br>FemaleTotal: 145	22.7 ± 3.29 y
Santos V et al.			212					
Byahatti S and Ingafou MS	751	5%	506	67.38%	187	329	MaleTotal: 100<br>FemaleTotal: 100<br>Male Impacted3M: 383;<br>Female Impacted3M:377	21.5±2.9
Lisboa, A et al.	1150	426	724	62.96%	only mandible	724	MaleTotal: 305<br>FemaleTotal: 419	

Gisakis IG et al.	940		861	91.6%	344	508	MaleImpacted: 395<br>FemaleImpacted: 466	36.69 years ± 13.69
Fernández JE et al.			1004		only mandible		MaleImpacted: 252<br>FemaleImpacted: 290	
Celikoglu M et al.	1161	243	444	38.2%	192	252	MaleImpacted: 108<br>FemaleImpacted: 336	22.8 ± 3.1
Hassan AH			577		228	349	MaleImpacted: 222<br>FemaleImpacted: 200	28.11 ± 7.25 years
Santos, L et al.							MaleImpacted: 16<br>FemaleImpacted: 44	
Nery, F et al.			70		only mandible	70	MaleImpacted: 12<br>FemaleImpacted: 34	
Chu FC et al.	7077	1399	3778	53.38%	600	3178		27.9y
Quek SL	2281		1385		306	1079	MaleImpacted: 302<br>FemaleImpacted: 384	26.5 **± 5**

Olasoji HO and Odusanya AS	9536	64	563	5.90%	158	405		
Yamaoka M et al.			115		68	47	MaleImpacted: 68<br>FemaleImpacted: 87	21-97y
van der Linden W et al.	3652	352	2872	78.64%	1135	1737		13-75y
Hattab FN et al.	814	114	194	28.2%				20.4y
Eliasson S et al.	1537	1039	1211	78.79%	477	734		
Haidar Z and Shalhoub SY	3681	319	1173	31.86%			MaleTotal: 624<br>FemaleTotal: 376	20-40y (mean 24y)

Cederhag J et al.	435			12% had a retained location, and 15% had a semi-retained location	only mandible		MaleTotal: 218 FemaleTotal: 224	
Al-Anqudi SM et al.	3287		1128		311	817	Male Total: 435; Female Total: 565; Male Impacted: 215; Female Impacted: 328	19-26
Eshghpour M et al.	2866	197	1397		only mandible	1397	Male total: 489; Female total: 944; MaleImpacted: 263; Female impacted: 608	25.44
Aitasalo K			587	71.3%	304	323	Male Total: 184; Female Total: 2222	
Al Feeli D & Sebaa Y			191		90	101	Male Total: 509; Female Total: 495	40.7±10.3
Dachi S & Howell F	2167		422				Male Total: 973; Male impacted: 183; Female Total: 1194; female impacted: 239	
Guo UC et al.							Male Total: 593; Male Impacted: 383; Female Total: 1306; Female impacted: 730	
Hassan AH							Male Total: 62; Male Impacted: 41; Female Total:59; Female Impacted: 30	23.89

Hatem M et al.	1200		843		371	472	Male Total: 156; Male Impacted 3M: 414; Female Total: 144; Female Impacted 3M: 429	29±6
Jan A et al.			1351		342	426	Male Impacted: 372; Female Impacted: 396	25-44
Jung YH & Cho BH	15196		1499		543	956		
Kabwe JK								
Kruger E et al.	2857		1801		814	987		
Perumal CJ	4512	2483			1203	2483	Male Total: 549; Male impacted: 1256; Female Total: 579; Female impacted: 1227	
Pillai AK et al.	3910	490	2210		460	1750	Male Total: 730; Female Total: 370	
Ramamurthy A et al.	2010	462			only mandible	462	Male Total: 516; Male Impacted: 267; Female Total: 489; Female Impacted: 195	

Venu K, et al.							Male Impacted: 109; Female Impacted: 59	22-30
Secic S et al.			1034		only mandible	1034	Male Impacted: 270; Female Impacted: 491	27± 9
Stanley HR et al.								
Shaddad, M et al.			903		417	486	Male total:127; Male Impacted:70; Female total: 449; Female Impacted: 273	
Alfadil L & Almajed E	3840		2240		929	1311	Male total:500; Male Impacted3M:1207; Female total: 514; Female Impacted3M:1033	30.67 ±8.98
Alhadi Y, et al.							Male Total: 174; Male Impacted: 61; female Total: 435; Female impacted: 174	
Arabion H, et al.							Male Total: 1586; Male Impacted: 358 ; female Total: 2046; Female impacted: 798	23.25±4.17
Bayoumi AM, et al.	2320		914		431	483	Male Total: 247; Male Impacted: 190; Female Total: 333; Female Impacted: 257	26.97

Gebeyehu T & Abaynew Y					15	43	Male Total: 113; Male Impacted: 33; Female Total: 178; Female Impacted: 31	
Hasan L et al.	3520	57	1100		428	672	Male Total: 498; Male Impacted: ; Female Total: 382; Female Impacted:	28.8
Idris A et al.			577		255	322	Male impacted: 263; Female impacted: 314	30.8
Ishwarkumar S et al.			709		309	400	Male Total: 129; Male Impacted:101 ; Female Total: 145; Female Impacted: 121	
Sivaramakrishnan SM & Ramani P	567	33	250		120	130	Male Total: 75; Male Impacted3M: 102 ; Female Total: 75; Female Impacted3M: 148	18-26
Sujon M et al.	11488		2433		649	1784	Male Total: 1491; Male Impacted: 684 ; Female Total: 1381; Female Impacted: 643	35.90± 10.76
Doni B & Shushma G							Male Total: 130; Male Impacted: 121 ; Female Total: 118; Female Impacted: 85	
Goyal S et al.			2331	492	171	321	Male Total: 454; Male Impacted3M:307 ; Female Total: 246; Female Impacted3M: 185	32.32 ±6.52

Jaiswal P								
Kumar SM et al.					60	139	Male Total: 85; Male Impacted:30 ; Female Total: 85; Female Impacted: 65	
Naraynsingh CN et al.			700		155	545	Male Total: 161; Male Impacted3M:262 ; Female Total: 247; Female Impacted: 438	
Omar L					205	372	Male Total: 517; Male Impacted:254 ; Female Total: 633; Female Impacted: 323	17-30
Pakravan AH et al.								20-68
Pechalova P et al.	3036	203	796					45.31±15.64
Qassadi TM et al.	4048		1164		398	766	Male Total: 550; Female Total: 462	31.51±7.918
Ren CG & Kumar BS							Male Total: 100; Male Impacted:31 ; Female Total: 100; Female Impacted: 29	

Shajahan S et al.								34.20±12.82
Taghain SM et al.	1044		86		51	35	Male Total: 138; Male Impacted:21 ; Female Total: 156; Female Impacted: 31	
Wagner MC	1431	569	176		28	148		26.64±8.04
Bhut M								17-50
Genc B et al.	1752		1036		308	728		29
Shareif MS et al.								18-50
SHIHAAB E NOOR S & RAJASEKAR A	2120	208	906					
Viqar S et al.							Male Total: 39; Male Impacted:22 ; Female Total: 51; Female Impacted: 36	

Yunus B & Tenrilili A							Male Total: 954; Male Impacted:286 ; Female Total: 1438; Female Impacted: 442	
Zhi Y et al.								18-30
Yacoub S et al.	2920		881		302	579	Male Total: 286; Male Impacted: 132 ; Female Total: 444; Female Impacted: 235	30

Author	Rx examination	3M eruption status	Pell and Gregory (level) Maxilla	Pell and Gregory (level) Mandible	Winter (angulation) Maxilla	Winter (angulation) Mandible
Abdelaziz, A A	OPG + cephalograms	**Maxila**: 340 impacted; 311 erupted **Mandible**: 337 impacted; 297 erupted	-	-	Mesioangular: 19<br>Distoangular: 99<br>Horizontal: 11<br>Vertical: 181	Mesioangular: 136<br>Distoangular: 17<br>Horizontal: 234<br>Vertical: 64
Poszytek & Górski	OPG	Erupted: 3788<br>Partially Impacted: 1013<br>Completely impacted: 423	-	-		
Adeola, O et al.	Periapical	-	-	-	-	Mesioangular:171<br>Distoangular: 74<br>Horizontal: 77<br>Vertical: 147
Naysmith, KE et al.	OPG	Maxila: 332 absent; 99 partially erupted; 450 erupted; 794 non-erupted<br>Mandible: 352 absent; 355 partially erupted; 256 erupted; 707 non-erupted	-	-	Mesioangular:187<br>Distoangular: 13<br>Horizontal: 7<br>Vertical: 20	Mesioangular:457<br>Distoangular: 20<br>Horizontal: 153<br>Vertical: 172
Shaari RB et al.	OPG		A: 9; B: 44; C: 135	A: 109; B: 127; C: 49<br>I: 26; II: 184; III:75	Mesioangular:22<br>Distoangular: 38<br>Horizontal: 6<br>Vertical: 105<br>Buccolingual: 16<br>Inverted: 1	Mesioangular:94<br>Distoangular: 19<br>Horizontal: 43<br>Vertical: 111<br>Buccolingual: 8<br>Inverted: 1 0
Salam S et al.	OPG					Mesioangular:232<br>Distoangular: 86<br>Horizontal: 56<br>Vertical: 114

Angelakopoulos N et al.	OPG	not impacted: 500	-	-	-	Mesioangular:325<br>Distoangular: 14<br>Horizontal: 72<br>Vertical: 125
Alassaf MS	OPG		-	-	Mesioangular:55<br>Distoangular: 64<br>Horizontal: 2<br>Vertical: 164	Mesioangular:186<br>Distoangular: 20<br>Horizontal: 55<br>Vertical: 126
Lamichhane NS et al.	OPG		-	A: 639; B: 69; C: 50<br>I: 602; II: 104; III:52	-	Mesioangular:344<br>Distoangular: 39<br>Horizontal: 126<br>Vertical: 249
Yildirim H, et al.	OPG	fully unerupetd: 98.4% maxilla; 70% mandible.	Partially erupted maxilla: 40.2% position A; 48.4% position B; 11.4% position C.	Partially erupted mandible: 67.3% position A; 31.6% position B; 1.2% position C	**Fully unerupted maxilla**: 56.6% vertical; 27.2% distoangular; 11.1% mesioangular; 1.7% horizontal; 0.3% inverted. **Partially erupted maxilla:** 68.9% vertical; 14.8% mesioangular; 13.1% distoangular; 3.3% horizontal.	**Fully unerupted mandible:** 58.2% mesioangular; 16.7% horizontal; 16.3% vertical; 6.6% distoangular; 0.01% inverted. **Partially erupted mandible:** 48.6% vertical; 27% mesioangular; 18.3% horizontal; 5.9% distoangular; 0.2% others
Alsaegh MA et al.	OPG		-	A: 151; B: 312; C: 223<br>I: 101; II: 508; III:77	-	Mesioangular:325<br>Distoangular: 132<br>Horizontal: 109<br>Vertical: 107

Santos KK et al.	OPG		-	A: 402; B: 423; C: 230<br>I: 246; II: 581; III:2228	-	Mesioangular:441<br>Distoangular: 264<br>Horizontal: 128<br>Vertical: 216<br>Inverted: 6
Ahmad P et al.	OPG			A: 706; B: 249; C: 67<br>I: 353; II: 501; III:168	Mesioangular:483<br>Distoangular: 159<br>Horizontal: 1<br>Vertical: 292	Mesioangular:359<br>Distoangular: 175<br>Horizontal: 158<br>Vertical: 330
Zaman UM et al.	OPG		-		-	Mesioangular:333<br>Distoangular: 149<br>Horizontal: 33<br>Vertical: 330<br>Others: 5
Haddad Z et al.	OPG		-	A: 684; B: 749; C: 167<br>I: 756; II: 820; III:24	-	Mesioangular:576<br>Distoangular: 111<br>Horizontal: 231<br>Vertical: 535<br>Buccolingual: 28<br>Others: 119
Ventä I et al.	OPG	erupted: 3548	-		Mesioangular:476<br>Distoangular: 176<br>Horizontal: 12<br>Vertical: 1745<br>Others: 17	Mesioangular:985<br>Distoangular: 433<br>Horizontal: 193<br>Vertical: 1598<br>Others: 9
KalaiSelvan S et al.	OPG		-	A: 326; B: 102; C: 30<br>I: 204; II: 229; III:25		
Rezaei, F et al.	OPG			A: 91; B: 118; C: 141<br>I: 223; II: 92; III:35	-	Mesioangular:121<br>Distoangular: 6<br>Horizontal: 87<br>Vertical: 118<br>Buccolingual: 17<br>Others: 1

Jain S et al.	OPG		não separa maxila e mandibula	A: 58; B:226; C: 118	Mesioangular:34<br>Distoangular: 38<br>Horizontal: 3<br>Vertical: 94	Mesioangular:121<br>Distoangular: 10<br>Horizontal: 69<br>Vertical: 33
Tassoker M et al.	OPG	Not impacted: 304			Mesioangular:45<br>Distoangular: 64<br>Horizontal: 0<br>Vertical: 12<br>Buccolingual: 1	Mesioangular:187<br>Distoangular: 3<br>Horizontal: 6<br>Vertical: 0<br>Buccolingual: 2
Sejfija Z et al.						
Helmi J et al.	OPG				Mesioangular:20.7%<br>Distoangular: 10.3%<br>Horizontal:13.5%<br>Vertical: 55.5%	Mesioangular:52.9%<br>Distoangular: 2.1%<br>Horizontal:30.7%<br>Vertical: 15.3%
Rashid, M et al.	CBCT		-	A: 41; B: 76; C: 18		
Kumar VR et al.	OPG		-	IA: 165; IB: 31; IC: 7; IIA: 33: IIB: 25; IIC: 13; IIIA: 1; IIIB:1	-	Mesioangular:146<br>Distoangular: 37<br>Horizontal: 12<br>Vertical: 78<br>Others: 3
Al-Dajani M et al.	OPG		A: 650; B: 67; C: 69	A: 714; B: 115; C: 108	Mesioangular:5<br>Distoangular: 69<br>Horizontal: 0<br>Vertical: 688<br>Transverse: 6	Mesioangular:144<br>Distoangular: 25<br>Horizontal: 71<br>Vertical: 665<br>Transverse: 0
Syed KB et al.	OPG					

El-Khateeb SM et al.	OPG		A: 18; B: 6; C: 42	A: 64; B: 64; C: 15; I: 25: II:111; III:7	Mesioangular:7<br>Distoangular: 2<br>Horizontal: 7<br>Vertical: 29<br>Buccolingual:10	Mesioangular:41<br>Distoangular: 2<br>Horizontal: 43<br>Vertical: 53<br>Buccolingual:4
Chicarelli da Silva, M et al.	OPG		A: 0; B: 55; C: 190	A: 142; B: 99; C: 178; I: 76: II:313; III:30	Mesioangular:82<br>Distoangular: 72<br>Horizontal: 0<br>Vertical: 88<br>Buccolingual:3	Mesioangular:127<br>Distoangular: 63<br>Horizontal: 77<br>Vertical: 143<br>Buccolingual:9
Hashemipour MA et al.	OPG		IA: 100; IB: 20; IC: 20; IIA: 218: IIB: 25; IIC: 14; IIIA: 18; IIIB:0; IIIC: 0	IA: 100; IB: 80; IC: 25; IIA: 292: IIB: 125; IIC: 55; IIIA: 14; IIIB:30; IIIC: 29	Mesioangular:82<br>Distoangular: 92<br>Horizontal: 46<br>Vertical: 188<br>Buccolingual:0<br>Other: 7	Mesioangular:362<br>Distoangular: 47<br>Horizontal: 220<br>Vertical: 116<br>Buccolingual:5<br>Other: 0
Topkara A and Sari Z	OPG		A: 0; B: 80; C: 68	A: 1; B: 102; C: 49	Mesioangular:10<br>Distoangular: 95<br>Horizontal: 7<br>Vertical: 35<br>Buccolingual:1	Mesioangular:99<br>Distoangular: 1<br>Horizontal: 39<br>Vertical: 10<br>Buccolingual:3
Santos V et al.	OPG		A: 71.8%; B: 18.6%; C: 9.6%	A: 66.7%; B: 24.8%; C: 8.5%; I: 66.3%; 25.7%; III:8%	Mesioangular:22.4%<br>Distoangular: 7.5%<br>Horizontal: 0.3%<br>Vertical: 69.8%	Mesioangular:61.5%<br>Distoangular: 7.5%<br>Horizontal: 6.8%<br>Vertical: 29.5%
Byahatti S and Ingafou MS	Clinical and OPG		A: 173; B: 95; C:104	A: 163; B:91; C:125	Mesioangular:22<br>Distoangular: 84<br>Horizontal:0<br>Vertical: 275	Mesioangular:158<br>Distoangular: 51<br>Horizontal:25<br>Vertical: 145
Lisboa, A et al.	OPG		-	A: 343; B 264; C: 117; I: 264; II:314; III:146	-	Mesioangular:328<br>Distoangular: 122<br>Horizontal: 77<br>Vertical: 194<br>Buccolingual:3

Gisakis IG et al.	OPG					
Fernández JE et al.	OPG				-	Mesioangular:36,85%<br>Vertical: 30,08%<br>Buccolingual:1,21%
Celikoglu M et al.	OPG				Mesioangular:27<br>Distoangular: 48<br>Horizontal: 2<br>Vertical: 135	Mesioangular:195<br>Distoangular: 3<br>Horizontal: 34<br>Vertical: 22
Hassan AH	OPG		A: 103; B: 110; C:15	A: 96; B: 235; C: 18	Mesioangular:39<br>Distoangular: 58<br>Horizontal: 17<br>Vertical: 113<br>Buccolingual:0<br>Other: 1	Mesioangular:117<br>Distoangular: 58<br>Horizontal: 96<br>Vertical: 72<br>Buccolingual:6<br>Other: 0
Santos, L et al.	OPG			AI: 2; AII: 13; AIII:7; BI: 11; BII: 20; BIII: 3; CI: 20; CII: 8; CIII: 2	Distal > prevalência	**38**: Mesio 30%; Disto 5%; Horizontal 45%; Vertical 20%; **48**: Mesio 36%; Disto 25%; Vertical 39%
Nery, F et al.	OPG			A: 19; B: 45; C: 6; I: 9; II:56; III:0	-	Mesioangular:40<br>Distoangular: 8<br>Horizontal: 7<br>Vertical: 15
Chu FC et al.	OPG					Mesioangular:1171<br>Distoangular: 313<br>Horizontal: 1508<br>Vertical: 134<br>Other: 52
Quek SL	OPG		A: 8; B: 180; C: 118	A: 62; B: 922; C: 95		Mesioangular:642<br>Distoangular: 106<br>Horizontal: 190<br>Vertical: 103<br>Buccolingual:6<br>Other: 6<br>Not applicable: 26

Olasoji HO and Odusanya AS	Clinical and periapical				vertical more frequent	mesioangular more frequent
Yamaoka M et al.	OPG					
van der Linden W et al.	OPG				Mesioangular:362<br>Distoangular: 153<br>Horizontal: 20<br>Vertical: 406<br>Transverse:77<br>Inverted: 0<br>Displaced: 117	Mesioangular:636<br>Distoangular: 336<br>Horizontal: 300<br>Vertical: 368<br>Transverse:40<br>Inverted: 0<br>Displaced: 57
Hattab FN et al.	OPG				-	Mesioangular:182<br>Distoangular: 19<br>Horizontal: 19<br>Vertical: 143
Eliasson S et al.	OPG	erupted: 326			vertical 39%	horizontal 41%
Haidar Z and Shalhoub SY	OPG	fully erupted: 2509				**both jaws**<br>Mesioangular:384<br>Distoangular: 96<br>Horizontal: 60<br>Vertical: 632

Cederhag J et al.	OPG		-	-	-	Mesioangular:50<br>Distoangular: 8<br>Horizontal: 22<br>Vertical: 35<br>Others: 3
Al-Anqudi SM et al.	OPG	Fully erupted: 2189; Impacted: 1128				Mesioangular:282<br>Distoangular: 267<br>Horizontal: 11<br>Vertical: 247<br>Others: 10
Eshghpour M et al.	OPG			A: 318; B: 892; C: 187; I: 510; II:677; III:210		Mesioangular:680<br>Distoangular: 84<br>Horizontal: 393<br>Vertical: 218<br>Others: 6<br>Buccolingual: 16
Aitasalo K	OPG					
Al Feeli D & Sebaa Y	OPG					
Dachi S & Howell F	OPG					
Guo UC et al.	OPG					
Hassan AH	OPG					

Hatem M et al.	OPG		A: 0; B: 146; C: 225	A: 222; B: 231; C: 19; I: 117; II: 343; III: 12	Mesioangular:63<br>Distoangular: 156<br>Horizontal: 0<br>Vertical: 149<br>Others: 1<br>Buccolingual: 2	Mesioangular:229<br>Distoangular: 77<br>Horizontal: 47<br>Vertical: 115<br>Others: 3<br>Buccolingual: 1
Jan A et al.	OPG				Mesioangular:84<br>Distoangular: 80<br>Horizontal: 11<br>Vertical: 167	Mesioangular:178<br>Distoangular: 7<br>Horizontal: 139<br>Vertical: 102
Jung YH & Cho BH	OPG		A: 0; B: 73; C: 470	A: 69; B: 467; C: 420	Mesioangular:137<br>Distoangular: 84<br>Horizontal: 5<br>Vertical: 258<br>Inverted: 7<br>Buccolingual: 52	Mesioangular:321<br>Distoangular: 9<br>Horizontal: 391<br>Vertical: 198<br>Inverted: 24<br>Buccolingual: 13
Kabwe JK	OPG					
Kruger E et al.	OPG				Mesioangular:400<br>Distoangular: 167<br>Horizontal: 0<br>Vertical: 247	Mesioangular:804<br>Distoangular: 18<br>Horizontal: 13<br>Vertical: 152
Perumal CJ	OPG				Mesioangular:140<br>Distoangular: 1061<br>Horizontal: 11<br>Vertical: 68<br>Inverted: 0	Mesioangular:478<br>Distoangular: 425<br>Horizontal: 185<br>Vertical: 115<br>Inverted: 0
Pillai AK et al.	OPG		A: 10; B: 90; C: 360	A: 960; B: 680; C: 110	Mesioangular:50<br>Distoangular: 70<br>Horizontal: 30<br>Vertical: 310<br>Other: 0	Mesioangular:580<br>Distoangular: 100<br>Horizontal: 340<br>Vertical: 720<br>Other: 10
Ramamurthy A et al.	OPG					

Venu K, et al.	OPG				Mesioangular:9<br>Distoangular: 7<br>Horizontal: 1<br>Vertical: 34<br>Unusual: 5	Mesioangular:41<br>Distoangular: 16<br>Horizontal: 28<br>Vertical: 22<br>Unusual: 5
Secic S et al.	OPG			IA: 437; IB:112; IC: 71; IIA: 122; IIB: 142; IIC: 35; IIIA: 9; IIIB: 7; IIIC:106		Mesioangular:206<br>Distoangular: 54<br>Horizontal: 92<br>Vertical: 673<br>Other: 4<br>Buccolingual: 5
Stanley HR et al.	OPG					
Shaddad, M et al.	OPG				Mesioangular:44<br>Distoangular: 136<br>Horizontal: 0<br>Vertical: 221<br>Buccolingual: 16	Mesioangular:309<br>Distoangular: 8<br>Horizontal: 93<br>Vertical: 45<br>Buccolingual: 31
Alfadil L & Almajed E	OPG		A:19; B:333;C:577	A:270; B:334; C:707; I:874; II:359; III:78	Mesioangular:75<br>Distoangular: 296<br>Horizontal: 9<br>Vertical: 525<br>Buccolingual: 20<br>Inverted: 4	Mesioangular:531<br>Distoangular: 41<br>Horizontal: 302<br>Vertical: 420<br>Buccolingual: 11<br>Inverted: 6
Alhadi Y, et al.						
Arabion H, et al.	OPG				Mesioangular:56<br>Distoangular: 216<br>Horizontal: 28<br>Vertical: 198<br>Other: 29	Mesioangular:308<br>Distoangular: 26<br>Horizontal: 84<br>Vertical: 182<br>Other: 29
Bayoumi AM, et al.	OPG		A: 24; B:176; C:231	A: 230; B:204; C:49; I: 219; II; 220; III:44	Mesioangular:70<br>Distoangular: 160<br>Horizontal: 5<br>Vertical: 190<br>Other: 3<br>Buccolingual: 3	Mesioangular:199<br>Distoangular: 26<br>Horizontal: 132<br>Vertical: 122<br>Other: 3<br>Buccolingual: 1

Gebeyehu T & Abaynew Y	OPG					
Hasan L et al.	OPG		A: 4; B: 56; C: 363	A: 208; B: 156; C: 313	Mesioangular:19<br>Distoangular: 150<br>Horizontal: 5<br>Vertical: 251<br>Inverted: 1<br>Buccolingual: 2	Mesioangular:184<br>Distoangular: 14<br>Horizontal: 51<br>Vertical: 407<br>Inverted: 3<br>Buccolingual: 13
Idris A et al.	OPG					
Ishwarkumar S et al.	OPG		A: 231; B:31; C:47	A: 65; B: 218; C: 117	Mesioangular:18<br>Distoangular: 51<br>Horizontal: 2<br>Vertical: 206<br>Buccolingual: 32	Mesioangular:210<br>Distoangular: 6<br>Horizontal: 88<br>Vertical: 96<br>Buccolingual: 0
Sivaramakrishnan SM & Ramani P	OPG				Mesioangular:43<br>Distoangular: 6<br>Horizontal: 10<br>Vertical: 35	Mesioangular:60<br>Distoangular: 9<br>Horizontal: 40<br>Vertical: 47
Sujon M et al.	OPG		A:56; B: 86; C:507	A:1110; B:232; C:442; I: 1112; II:240; III:432	Mesioangular:147<br>Distoangular: 363<br>Horizontal: 25<br>Vertical: 48<br>Others: 66	Mesioangular:652<br>Distoangular: 1<br>Horizontal: 565<br>Vertical: 521<br>Others: 45
Doni B & Shushma G	OPG					
Goyal S et al.	OPG				Mesioangular:31<br>Distoangular: 82<br>Horizontal: 2<br>Vertical: 40<br>Others: 0<br>Buccolingual: 16	Mesioangular:173<br>Distoangular: 66<br>Horizontal: 28<br>Vertical: 50<br>Others: 2<br>Buccolingual: 2

Jaiswal P	OPG					
Kumar SM et al.	OPG				Mesioangular:12<br>Distoangular: 15<br>Horizontal: 5<br>Vertical: 28	Mesioangular:39<br>Distoangular: 11<br>Horizontal: 28<br>Vertical: 61
Naraynsingh CN et al.	OPG					
Omar L	OPG					
Pakravan AH et al.	OPG			A:109; B:61; C:10; I: 107; II:49; III:24		Mesioangular:81<br>Distoangular: 24<br>Horizontal: 15<br>Vertical: 60
Pechalova P et al.	OPG					Mesioangular:373<br>Distoangular: 76<br>Horizontal: 92<br>Vertical: 234<br>Others: 6<br>Buccolingual: 15
Qassadi TM et al.	OPG		A: 46; B: 120; C: 232	A: 440; B: 22; C: 104; I: 239; II: 498; III: 30	Mesioangular:58<br>Distoangular: 192<br>Horizontal: 4<br>Vertical: 142	Mesioangular:235<br>Distoangular: 133<br>Horizontal: 73<br>Vertical: 323
Ren CG & Kumar BS	OPG					

Shajahan S et al.	OPG					Mesioangular:93<br>Distoangular: 11<br>Horizontal: 64<br>Vertical: 30
Taghain SM et al.	OPG		A: 0; B: 2; C: 33	A:19; B:15; C:17; I: 9; II:40; III:2	Mesioangular:5<br>Distoangular: 11<br>Horizontal: 0<br>Vertical: 18	Mesioangular:19<br>Distoangular: 0<br>Horizontal: 13<br>Vertical: 19
Wagner MC	OPG					
Bhut M	OPG			A:104; B:183; C:31; I: 102; II:152; III:64		Mesioangular:144<br>Distoangular: 45<br>Horizontal: 102<br>Vertical: 21 Other: 6
Genc B et al.	OPG					
Shareif MS et al.	OPG					
SHIHAAB E NOOR S & RAJASEKAR A	OPG					
Viqar S et al.	OPG					

Yunus B & Tenrilili A	OPG					
Zhi Y et al.	OPG					
Yacoub S et al.	OPG		A: 59; B: 44; C: 199	A: 276; B: 196; C: 107; I: 67; II: 442; III: 70	Mesioangular:45<br>Distoangular: 91<br>Horizontal: 0<br>Vertical: 157<br>Others: 3<br>Buccolingual: 6	Mesioangular:185<br>Distoangular: 12<br>Horizontal: 114<br>Vertical: 264<br>Others: 0<br>Buccolingual: 4

Author	Pathology 3M	Pathology adjacent 2M
Abdelaziz, A A	excluded	excluded
Poszytek & Górski	-	2M caries: 133; Root resorption: 89; Bone loss: 464
Adeola, O et al.	Pericoronaritis: 452; Pulpitis: 15; Abcess: 1	-
Naysmith, KE et al.	-	-
Shaari RB et al.		
Salam S et al.		

Angelakopoulos N et al.	-	
Alassaf MS		
Lamichhane NS et al.		
Yildirim H, et al.	Mesial carie 3M: 37.9% (33.6% maxilla; 4.3% mandible); Caries on both: 25.8% (12.3% maxilla; 13.5% mandible); No caries: 97.7% (34.4% maxilla; 63.3% mandible)	Distal carie 2M: 38.6% (19.7% maxilla; 18.9% mandible)
Alsaegh MA et al.	Carie in 3M: 24 (more frequent in mesioangular, level A and B, Class I and II)	Distal carie 2M: 102

Santos KK et al.		
Ahmad P et al.	Dental caries: 301; periodontal pocket: 229; dentigerous cyst: 9; 2M root resorption: 5	
Zaman UM et al.		
Haddad Z et al.	3M pathological lesions: 119	2M root resorption: 252; 2M distal caries: 195
Ventä I et al.		
KalaiSelvan S et al.		2M distal caries: 90
Rezaei, F et al.		

Jain S et al.		
Tassoker M et al.		
Sejfija Z et al.	Enlarged pericoronar gap: 15	Caries of impacted 3M and/or 2M: 33 Periodontal bone loss 2M >5mm: 71 Root resorption 2M: 307
Helmi J et al.		
Rashid, M et al.		Distal caries 2M: 58 (43%)
Kumar VR et al.		
Al-Dajani M et al.		
Syed KB et al.		Distal carie 2M: 377 (39%)

El-Khateeb SM et al.		
Chicarelli da Silva, M et al.		
Hashemipour MA et al.		
Topkara A and Sari Z		
Santos V et al.		
Byahatti S and Ingafou MS		
Lisboa, A et al.		

Gisakis IG et al.	Carie of impacted/adjacent tooth: 43; increase pericoronal space: 49; orthodontic complications: 44	Periodontal bone loss adjacent tooth: 117; 2M rooth resorption: 81
Fernández JE et al.		
Celikoglu M et al.	coronal radiolucency in 3M: 6	2M distal caries: 10; 2M root resorption: 17; 2M distal bone loss: 13
Hassan AH		
Santos, L et al.		
Nery, F et al.		
Chu FC et al.	**Mandible**: Caries 3M: 80	Mandible: periodontal disease 2M: 282; Carie2M: 234
Quek SL		

Olasoji HO and Odusanya AS		
Yamaoka M et al.		
van der Linden W et al.	**2872 had pathology** Caries3M: 204; Alveolar bone decrease: 140; Coronal radiolucency:133; Periapical radiolucency: 67; Osteitis:: 46; supernumerary teeth: 16; root resorption: 26	Caries2M: 1227
Hattab FN et al.		
Eliasson S et al.	Widened pericoronal space, resorption2M and severe marginal bone loss: 25 in maxillary Impacted 3M and 59 in mandibular impacted 3M	
Haidar Z and Shalhoub SY		

Cederhag J et al.		
Al-Anqudi SM et al.	Radiolucency >2.5mm: 116; caries: 33	
Eshghpour M et al.		
Aitasalo K		
Al Feeli D & Sebaa Y		
Dachi S & Howell F		
Guo UC et al.		
Hassan AH		

Hatem M et al.		
Jan A et al.	Caries of impacted 3M: 213; Periodontal pocket: 1133; Follicular hyperplasia/dentigerous cyst: 4	Caries 2M: 320; Abcess: 417; Root resorption 2M: 1
Jung YH & Cho BH		
Kabwe JK		
Kruger E et al.		
Perumal CJ		
Pillai AK et al.		
Ramamurthy A et al.		

Venu K, et al.		
Secic S et al.	3M or 2Mcaries: 174; Periodontal pocket3M:134; Dentigerous cyst:5; 2M root resorption:2	
Stanley HR et al.		
Shaddad, M et al.		
Alfadil L & Almajed E		
Alhadi Y, et al.		
Arabion H, et al.		
Bayoumi AM, et al.		

Gebeyehu T & Abaynew Y		
Hasan L et al.		
Idris A et al.		
Ishwarkumar S et al.		
Sivaramakrishnan SM & Ramani P		
Sujon M et al.		
Doni B & Shushma G		
Goyal S et al.		

Jaiswal P		
Kumar SM et al.		
Naraynsingh CN et al.	Associated cyst: 8; Caries:60	Root resorption: 4; Caries:74
Omar L		
Pakravan AH et al.		
Pechalova P et al.		
Qassadi TM et al.		
Ren CG & Kumar BS		

Shajahan S et al.		
Taghain SM et al.		
Wagner MC		
Bhut M		
Genc B et al.	Pericoronaritis: 178; Caries:73; Periodontitis: 43; Abcess/cysts: 35	Caries: 33; root resorption: 21
Shareif MS et al.		
SHIHAAB E NOOR S & RAJASEKAR A		
Viqar S et al.		

Yunus B & Tenrilili A		
Zhi Y et al.		
Yacoub S et al.	Pericoronaritis: 22; Caries: 46; Cysts: 16	Root resorption: 12; Caries: 103
